# Seeing Conflict: Identifying Self-Control Conflict Attenuates Early Allocation of Visual Attention to Positive Stimuli

**DOI:** 10.1177/17470218251389519

**Published:** 2025-10-15

**Authors:** Julia Vogt, Yasmene Bajandouh, Louisa Butler

**Affiliations:** 1 School of Psychology and Clinical Language Sciences, University of Reading, UK

**Keywords:** spatial attention, positive, self-control, temptations, reward, emotion

## Abstract

Positive information such as temptations or stimuli related to reward attracts attention. This attentional bias is often considered to be habit-like and, consequently, to contribute to self-control problems. In contrast, we document across three studies how attention allocation to positive stimuli is attenuated when observers identify a conflict between positive information and a more important current goal. In study 1, students attended away from leisure-related temptations in the presence of studying reminders, however, they paid more attention to such positive, tempting stimuli when conflict was less salient such as when planning to engage in more leisure activities. Study 2 and 3 manipulated experimentally whether a conflict is identified and tested the effects of conflict identification on attention allocation. In study 2, students attended away from a positive temptation when they were prompted to consider it as detrimental to their studying goals but not when they reflected on how it might support their studying goals. This supports our assumption that activating a more important goal by itself does not cause attenuation of attention but the identification of a conflict with a goal. In study 3, reward-related information was attended to, but attention was attenuated when participants were instructed that the stimuli would temporarily harm and thus be in conflict with a more important goal. This effect was visible even without allowing participants to forget about the association with reward. In sum, malleable perceptions of conflict between positive information and important goals shape how observers orient to such information.

## Introduction

Have you ever struggled to *not* pay attention to a tempting item such as a delicious food item on display in a store or a social media platform that calls to be checked for updates? In many daily situations, people encounter items that have a strong and positive affective value; such items have been described as temptations, desires, or rewards (Hofmann & Van Dillen, 2012; Pearson et al., 2022; Pool et al., 2016; but see Chiew, 2021). These items are often considered to evoke instant positive affect and, consequently, to attract attention (see Hardman et al., 2021; Pool et al., 2016; Vogt et al., 2020; Watson et al., 2019, for overviews). This attentional bias is thought to contribute and underlie self-control failures, because it enhances craving in addition to being quick and hard to control (Nikolaidou et al., 2019; Papies et al., 2008; Pearson et al., 2022; Watson et al., 2019). In contrast, in the present paper, we ask whether and when people will be inattentive to such positive stimuli, even at early stages of attention allocation. Particularly, we aim to test whether the identification of a conflict with a more important goal (e.g., considering social media as an obstacle to writing a paper) impacts attention to positive stimuli and causes people to display inattention to these items already at early stages of attention allocation.

Our work is based on theories that consider self-control and attention allocation as automatic but flexible (Cole & Balcetis, 2021; Fishbach & Shen, 2014; Pessoa & Adolphs, 2010; Vogt et al., 2020). According to this perspective, early visual processes might serve the pursuit of higher-order goals. We also ask when self-control is activated (Goschke & Job Sutnar, 2023; Hennecke & Bürgler, 2023; Inzlicht et al., 2020; Kotabe & Hofmann, 2015; Myrseth & Fishbach, 2009; Werner & Ford, 2023). Specifically, we will investigate whether the identification of a self-control conflict will attenuate attention towards positive stimuli. We argue that recognizing a conflict between a positive stimulus and a higher order goal is sufficient to attenuate attention allocation. This is in line with recent calls to reconsider the role of inhibition as the major determinant of self-control success or failure (e.g., Hennecke & Bürgler, 2023; Werner et al., 2022).

### Allocation of Visuospatial Attention to Motivationally Relevant Events

Attention determines the processing of any information an observer encounters in their environment (Desimone & Duncan, 1995; Yoo et al., 2022). A crucial component of attention is the allocation of visuospatial attention (cf. Lee & Pitt, 2022; Posner, 1980; Wilterson & Graziano, 2021). Attention to location is a spotlight that enhances processing of stimuli at that location (Posner, 1980; but see Lee & Pitt, 2022). We will examine this component of attention in contrast to other accounts that have investigated different so-called attentional processes in self-control conflicts such as the accessibility of goals and temptations (Mann & Ward, 2007) or response interference (Van Dillen et al., 2013; see Luck & Vecera, 2002, for a classification of attentional processes). Allocation of visual spatial attention takes place earlier than the latter processes; it is predictive of people’s emotional states, judgements and choices, and their behaviour (e.g., Van Bockstaele et al., 2014; Wilterson & Graziano, 2021; Yang & Krajbich, 2023).

Motivationally relevant events attract visuospatial attention (Abado et al., 2020; Yiend, 2010). Prominent theories suggest that this mechanism developed as an adaptive response serving the survival or reproduction motive. Indeed, attention is biased towards events with a strong emotional value (Yiend, 2010). For instance, events with a negative or threat value attract attention such as dangerous animals or angry faces (see Abado et al., 2020, or Yiend, 2010, for overviews). Events with a positive value also attract attention such as beautiful people (J. Maner et al., 2003), or babies (Brosch et al., 2007). Importantly, positive events are not inferior to negative events in attracting attention (Brosch et al., 2008; Vogt et al., 2008). Automatic attentional bias is not limited to stimuli with an inborn emotional value but extends to events with a learned emotional value. For instance, weapons or outgroups attract attention (Brosch & Sharma, 2005; Donders et al., 2008). Relatedly, participants pay attention to food that they like (Tapper et al., 2010; Werthmann et al., 2016), social media content (e.g., Nikolaidou et al., 2019) or stimuli representing self (S. J.Cunningham & Turk, 2017). Similarly, addiction has been associated with attentional bias to substances (Field et al., 2016). In a seminal study, Roskos-Ewoldsen and Fazio (1992) showed how people attend quickly to stimuli that are associated with strong positive attitudes suggesting that attention allocation allows people to become aware of positive opportunities in their environment. It appears to be sufficient to learn this value in the experimental situation for both threatening stimuli (e.g., through fear conditioning, Notebaert et al., 2011) and for monetary and symbolic reward (e.g., Walsh et al., 2021; see Watson et al., 2019, for an overview; also see Chiew, 2021, for arguments to consider positive affect and reward as distinct).

These accounts can explain why positive events grab attention, but they fail to explain why attention to positive stimuli might vary such as when people are inattentive to positive events (e.g., Brown et al., 2018; Godara et al., 2020; Papies et al., 2008). Failures to find attention to positive stimuli or incoherent findings such as in substance abuse and obesity have therefore led to calls for consideration of contextual factors to explain when and why attentional bias to positive events occurs (see Field et al., 2016; Hardman et al., 2021). For instance, Hardman et al. (2021) concluded that state rather than trait motivation might be a stronger factor in attention allocation than previously assumed.

This aligns with findings showing that the current relevance of stimuli shapes attention. For instance, making neutral objects or words relevant to a current goal pursued in a parallel task in the experimental situation even when these stimuli were only shown shortly and attention allocation was not useful for achieving the goal (Vogt et al., 2020, 2022; Wieber & Sassenberg, 2006). Importantly, events relevant to current goals even seem to override attention to events with an innate or (over)learned affective value (Brown et al., 2018, 2020; Correll et al., 2014; Forrest et al., 2022; Godara et al., 2020; Vogt et al., 2017). As an example, making neutral words or images relevant to a goal in the experimental situation overrides attention to various threats (Brown et al., 2020; Forrest et al., 2022; Vogt et al., 2013, 2022), to positive stimuli such as the self (S. Cunningham et al., 2022), and to alcohol for heavy drinkers or alcoholics (Brown et al., 2018; Godara et al., 2020). In the present paper, we extend this approach by asking how people’s perceptions of how temptations facilitate or interfere with an important goal impact attention.

### Motivated Attention from a Self-Control Perspective

Self-control conflicts can be considered as a form of *inherent* conflicts between two goals, specifically, between immediate, relatively short-term interests versus long-term, important goals (e.g., relaxation now versus a paper later; see Kung and Scholer (2021) for on an overview of different goal conflicts). Theories of self-control suggest that attention away from positive stimuli that represent temptations supports successful self-control (Inzlicht et al., 2014; Kaplan & Berman, 2010; Mann & Ward, 2007; Metcalfe & Mischel, 1999; Werner & Ford, 2023). For instance, children who could wait longer for a second marshmallow or pretzel, looked away from the marshmallows or pretzels and paid more attention to other stimuli in the room (Rodriguez et al., 1989). In a similar vein, people who paid less attention to attractive people were more likely to remain faithful in their relationships (McNulty et al., 2018). Indeed, people seek to distance themselves from temptations so that they are less visible to them (Cole et al., 2021). In sum, this research suggests that successful self-control is associated with attention away from temptations (but see DeWall et al., 2011).

When is attention to positive stimuli attenuated? People appear to *not* show positive automatic reactions towards positive, tempting stimuli when relevant higher order goals such as studying or health-related goals are activated (Fishbach et al., 2003; Roefs et al., 2006), for instance, they do not like, think about, approach, or pay attention to temptations such as unhealthy but delicious food or activities that could distract them from studying (Fishbach & Shah, 2006; Fishbach et al., 2003; Kleiman et al., 2016; Papies et al., 2008; Sah et al., 2021). For instance, Papies et al. (2008) measured how restrained eaters attend to food items after exposure to food. Initially, exposure to food caused an attentional bias to food. However, this bias was absent when a dieting goal was activated as part of the experimental procedure. Similarly, participants in committed relationships were faster to targets appearing in the *opposite* location of images of attractive people when they were reminded of their relationships causing the authors to suggest that they attend away from these images (J. K. Maner et al., 2009; McNulty et al., 2018). However, it is unclear whether this reflects a general response speeding or attentional avoidance because the authors do not compare responses to trials where targets appeared in the same location as the images (see Koster et al., 2004, for a discussion of the interpretation of these effects). Further, given the evidence above, it is unclear whether the activation of any goal would have been sufficient to reduce attention to highly positive stimuli (see Correll et al., 2014).

Here, we argue that activating higher-order goals by itself might not be sufficient to induce attentional attenuation, but that people must identify a conflict, that is, they expect a positive stimulus to interfere with a higher-order goal. This is important because perceptions of whether an activity or stimulus is interfering with or facilitating a goal are malleable. For instance, consuming a positive item might be considered adaptive when people think of how this serves legitimate (other) higher-order goals directly, for instance, enjoying social media can serve the goal of connecting with others, or indirectly, such as considering how enjoying unhealthy food items or enjoyable activities can help people to maintain a difficult goal and also well-being (Prinsen et al., 2019). Indeed, people who are good at self-control balance fun and duty (Becker et al., 2024; Jia et al., 2018). Thus, people might only *not* attend to positive stimuli when they see a conflict with a higher-order goal. This is in keeping with views arguing that the perception of and behaviour towards temptations change depending on the current context (cf.Berkman et al., 2017; Fishbach et al., 2003).

Recent research has therefore highlighted the relevance of identifying self-control conflicts (Goschke & Job Sutnar, 2023; Kotabe & Hofmann, 2015; Myrseth & Fishbach, 2009). By conflict identification, these accounts refer to the notion that a positive stimulus, that is, a temptation, and a goal appear incompatible (e.g., perceiving time on social media as harming the goal of writing or reading a paper) instead of compatible (e.g., perceiving time on social media as a goal that can be combined (e.g., alternated) with the goal of writing a paper) in contrast to accounts that refer to the affective and often aversive experience of a (response) conflict (e.g., Becker et al., 2019; Gillebaart & De Ridder, 2015). Conflict identification can be biased, for instance, asking participants to reflect on (assumed) progress towards a goal causes them to indulge (Fishbach & Dhar, 2005). Similarly, people might fail to perceive a conflict when indulging is perceived as a negligible exception (Myrseth & Fishbach, 2009). In the present work, we measured and manipulated whether a positive stimulus or activity appears to serve or harm a focal goal to test whether this perception guides attention allocation. Therefore, the present experiment served to test whether identifying conflict is sufficient to evoke a self-control response (i.e., attenuated attention towards temptations) even when measuring an early and automatic response.

### Overview of the Current Studies

The present studies investigated whether attentional bias to positive, rewarding stimuli will be attenuated when people consider a positive stimulus to conflict with a more important goal. We used cueing paradigms, that is, a dot probe task (MacLeod et al., 1986; study 1) and spatial cueing tasks (Fox et al., 2001; Posner, 1980; studies 2, 3) to examine the orienting of attention. In these tasks, one (spatial cueing task) or two (dot probe task) cue stimuli are presented on the screen, immediately followed by a target. If individuals selectively orient to a cue category, responses will be faster to targets at the location previously occupied by that cue but slower when the target is in the opposite location. In the spatial cueing task, cues that appear in the subsequent target location are called ‘valid’, whereas cues in the opposite location are called ‘invalid’. In the dot probe task, valid cues are commonly referred to as ‘congruent’ and invalid cues as ‘incongruent’.

In study 1, we tested attention allocation to images representing leisure-related activities (e.g., YouTube) when presented in parallel with reminders of study-related items. The study was done to replicate a similar pilot study and allowed us to test whether attentional bias to positive stimuli can indeed be attenuated. We also measured participants’ current motivation to engage in leisure and studying activities to gain first insights into the underlying mechanism. We reasoned that being motivated to pursue leisure activities could indicate that participants do not perceive a conflict *at this point in time* (e.g., otherwise they might report to be more motivated to invest in studying activities) and should thus promote attention to temptations, whereas motivation to engage in studying activities should enhance conflict perception and attenuate attention to temptations.

To address the role of conflict identification directly, studies 2 and 3 manipulated experimentally whether a conflict is identified or not. In study 2, we measured attention to a positive stimulus that was a common temptation for students, that is, Facebook, and directly manipulated whether participants perceive Facebook as conflicting or not with their studying goals before the attentional measure. This allowed us to test whether the perception of conflict and not the mere activation or commitment to a higher order goal or simply high self-control aptitude impacts attention allocation. In study 3, we tested whether participants can flexibly switch attention allocation to positive stimuli associated with reward depending on whether they were perceived as conflicting with a more important goal in a within-subjects design. This allowed us to test whether attention would be allocated to positive stimuli in a flexible way even in the same experimental situation. All data and materials can be found here: https://osf.io/2c8ef/.

## Study 1

This experiment tested whether students display an attentional bias towards study- or leisure-related items while also measuring their motivation to pursue leisure and studying activities. We reasoned that motivation to engage in leisure activities should facilitate attention to temptations as participants should not perceive a conflict at this very moment, whereas motivation to engage in studying activities should make a conflict salient and attenuate attention to temptations. The experiment was based on a pilot study with 46 participants following a design very similar to the one reported below except for some of the questions asked after the experiment. This study delivered a significant effect of congruency to the temptation, *F*(1,45) = 32.39, *p* < .001, η_*p*_^2^ = .419, 95% CI [−17.82, −8.51], indicating faster reaction times on temptation incongruent trials than on temptation-congruent trials. This reflects attention away from temptations.

### Method

#### Participants

Fifty students at the University of Chicago (19 women; *M*_age_ = 20.10 years, *SD*_age_ = 1.63 years) participated for a monetary reward of $5. Sample size was based on the pilot study that suggested a sample size of 28, using Cohen’s *d* = 0.84 at an alpha of .05 and a power of 0.8, but we raised the sample size to be able to conduct correlational analyses. Students studied a variety of majors such as Economics, Mathematics, English Literature, and many others. Ethical approval was obtained from the Social and Behavioral Sciences Institutional Review Board at the University of Chicago (H11249, Attention & Self-Control).

#### Apparatus and Materials

##### Pictorial Cues

We used 10 images of study-related materials and 10 images of leisure-related materials as cues in the attention task. These were two different images of five study-related materials and five leisure-related materials. As study-related materials, we used images of open textbooks, notes, and handouts from students at the University of Chicago. As leisure-related materials, we used images of a Facebook profile, Hulu, YouTube, Magazines and a TV show. We identified all items with surveys that were filled out by participants from the same participant pool. In this survey, we asked participants about typical distractions and temptations when studying. In the pilot study, 46 participants rated study-related items as highly representative of studying-related activities on a 7-point Likert scale (*M* = 6.09; *SD* = 1.08), and temptation-related activities as representative of temptations when studying on a 7-point Likert scale (*M* = 5.21; *SD* = 2.21). Ten neutral images were used in the neutral trials. These images did show tools, household items, or furniture.

##### Attention Task

**Figure 1.**
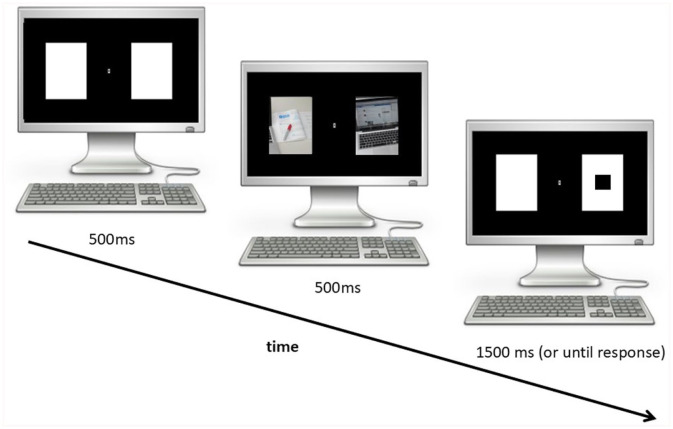
Schematic overview of a trial in the attention task. A trial started with the presentation of a fixation screen for 500 ms, followed by the presentation of two cue images for 500 ms. Then a probe (black square) was presented. Participants had to indicate the location of the probe. A trial ended after a response was registered or 1,500 ms had elapsed since the onset of the probe. The following trial started 200 ms after a response was registered or the presentation of the probe had ended. The right cue in this example consists of an image representing a temptation, and the left cue represents studying.

A trial in the attention task started with a fixation screen consisting of a white fixation cross (0.80 × 0.80 cm) in the middle of the black screen (see Figure 1). Two white rectangles (3.61 × 4.94 cm) were presented left and right of the fixation cross. The centre of the rectangles was placed 3 cm left and right of fixation. After 500 ms, two image cues (3.61 × 4.94 cm) appeared for 500 ms in the rectangles. A probe (black rectangle, 0.85 × 0.93 cm) appeared in one of the rectangles immediately after cue offset. Participants had to indicate the location of the probe by pressing one of two keys (‘a’, ‘l’) with left and right index finger on a QWERTY keyboard. A trial ended after a response was registered or 1,500 ms had elapsed since probe onset. The following trial started after 200 ms.

We included ‘letter trials’ to reduce strategic monitoring of one area of the screen and force people to attend to the fixation cross. On these trials, the fixation cross was followed only by a randomly selected letter for 150 ms in the location of the fixation cross. Participants were instructed to provide the letter presented. The following trials started after 1,100 ms.

#### Procedure

The experiment was presented on a Dell Dimension 5000 computer with an 85 Hz, 17-inch CRT monitor using the INQUISIT Millisecond software package (Inquisit 2.0, 2005). Participants were seated approximately 60 cm from the computer screen. During the entire experiment, participants read the instructions on the screen. Participants started with a practice phase of the attention task of 71 trials with a different set of neutral images as cues.

The test phase of the attention task consisted of 164 trials. These were 80 trials showing 2 neutral images, and 80 trials presenting 1 leisure-related and 1 study-related image. Participants also completed four letter trials. Each image category was presented equally often in the left and right location and predicted the probe location correctly on half of the trials. The task was programmed in such a way that each image of an image category had to be shown once before an image was shown again. The order of trials was determined randomly and for each participant separately.

Hereafter, we asked participants several questions to check the evaluation of the materials and to understand their habitual self-regulation patterns towards leisure- and studying-related activities, and, importantly, their current motivation for the upcoming weekend. Specifically, we asked participants to indicate in how far they experienced each of the five leisure-related activities as temptations when studying (1 = *not at all*; 7 = *very much*). We then asked them to indicate how important it is to them to engage in leisure-related activities (1 = *not at all*; 7 = *very much*). To measure their current motivation to engage in leisure- and study-related materials, we asked them to indicate the number of hours they plan to spend *during the next weekend* on both activities.

In order to measure goal commitment, we asked them how important it is to them to get good grades (1 = *not at all*; 7 = *very much*), and how difficult it is for them to get good grades (1 = *not at all*; 7 = *very much*) and to finish their coursework (1 = *not at all*; 7 = *very much*). We also asked for their last numerical Grade Point Average ( GPA). To get a better impression of the composition of our sample we also asked for their nationality, age, gender, participation in classes, major, and year of study. We also asked them which kind of studies they would prefer if they would come to the laboratory again in the upcoming days (1 = *Task that trains cognitive skills*; 2 = *Easy, engaging study*) in another attempt to measure their motivation to engage in study- and fun-related activities. Finally, we asked them what the study was about in their opinion.

### Results

#### Description of Sample

Participants were highly motivated to get good grades (*M* = 7.56; *SD* = 1.51). Furthermore, they had relatively high average GPAs (*M* = 3.35; *SD* = 0.45) and reported medium levels of difficulty to achieve good grades (*M* = 4.70; *SD* = 1.25) or to finish their coursework (*M* = 4.08; *SD* = 1.54). Still, leisure-related activities were important to them as well, *M* = 5.52, *SD* = 0.91. Participants reported experiencing leisure-related activities as moderate temptations when studying, *M* = 3.70, *SD* = 1.28. Though averaging these ratings was justified (α = .738), these ratings varied for single items with Facebook being described as the strongest temptation when studying, *M* = 5.28; *SD* = 2.01. The amount of hours they wanted to spend on studying (*M* = 10.67 hr; *SD* = 4.85 hr) and leisure activities (*M* = 11.94; *SD* = 6.13) during the next weekend after the study did not differ, *t*(48) = 1.02, *p* = .31, *d* = 0.15.

#### Main Analyses

##### Attention Task

Trials with errors were removed (1.36%). We excluded reaction times faster than 150 ms and slower than 631 ms, which is the sum of the sample’s mean Reaction Times (RT) plus three times the sample’s standard deviation (*SD*) of the RT (1.5%). We excluded the data of three participants who represented visual outliers upon visual inspection when checking the correlations. Their data points were more than 2.33 times the sample’s *SD* away from the sample’s mean. None of the findings below change when these participants are included.

To test our hypotheses, we run a repeated measures ANOVA (Analysis of Variance) with congruency to the temptation (congruent, incongruent) as within factor. A trial was designated as congruent if the probe replaced the leisure-related stimulus and as incongruent if the probe replaced the study-related picture. Most importantly, this analysis revealed a significant effect of congruency, *F*(1,46) = 90.19, *p* < .001, η_*p*_^2^ = .66. Importantly, this effect reflected faster reaction times on temptation incongruent trials than on temptation-congruent trials, reflecting attention away from temptations and towards study-related items.

**Figure 2.**
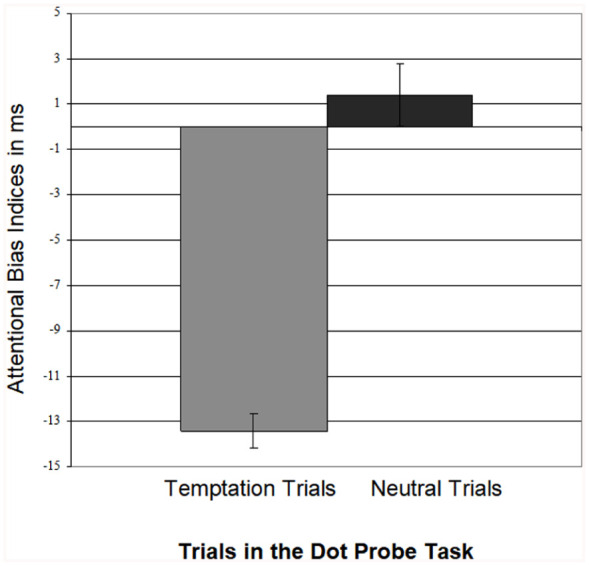
Mean dot probe reaction time difference scores (i.e., temptation/neutral incongruent trials minus temptation/neutral congruent trials). Error bars represent one standard error of the mean.

To further explore this effect, we calculated an attentional bias index by subtracting reaction times on temptation congruent trials from reaction times on temptation incongruent trials (see also Figure 2). Positive indices indicate attention towards temptations, negative indices indicate attention towards study-related items. We also calculated such an index for neutral trials by randomly assigning trials to be congruent or incongruent to create a baseline. Importantly, the attentional bias index for temptation-studying trials was also significantly more negative than the index for neutral items, *t*(46) = −6.57, *p* < .001, 95% CI [−16.52, −8.78], *d* = 0.96.

We also calculated an average score of all neutral trials as a form of baseline and compared it to both temptation congruent and temptation incongruent trials. Participants reacted slower on temptation congruent trials (*M* = 403.74; *SD* = 37.41) than on this baseline (*M* = 390.72 ms; *SD* = 37.08 ms), *t*(46) = 10.81, *p* < .001, 95% CI [10.60, 15.45], *d* = 0.1.58, but reaction times on temptation incongruent trials (*M* = 390.32 ms; *SD* = 37.49 ms) and this baseline did not differ, *t*(46) = −0.28, *p* < .783 [−3.26, 2.48], *d* = 0.04.

We then investigated whether the attentional bias index correlated with participants’ current motivation to engage in studying or leisure-related activities. As expected, the analyses revealed that more attention to leisure-related activities was associated with more hours planned for leisure during the next weekend after the experiment, *r*(46) = 0.32, *p* = .032 (Spearman’s ρ [46] = .302, *p* = .041). To check the robustness of this correlation, we conducted partial correlations controlling for the various indicators of attitudes towards goals and activities and successful indicators of self-control, that is, the importance of studying and leisure, the difficulties to achieve good grades and to finish their coursework, their GPA, and the average score of experiencing leisure-related activities as temptations when studying, *r*(38) = .34, *p* = .032. However, the correlation between the attention index and the hours planned for studying and the choice of future study was not significant, *p*s >.150. A closer look at participants’ responses regarding which type of study they would prefer to take part in the near future revealed that the question might not have measured motivation towards studying-related activities. Participants with a higher GPA were significantly more likely to choose easy and engaging studies, Spearman’s ρ (46) = .359, *p* = .014.

##### Secondary Analyses

As described above, participants described the selected leisure activities as medium temptations when studying. The scores might reflect participants’ high level of self-control, for instance, they might like the items but experience little conflict because they are proficient at self-control (e.g., Fishbach & Shen, 2014). However, we also tested whether participants attended away from image cues portraying Facebook because this was the leisure-related item that participants described as most tempting (*M* = 5.28; *SD* = 2.01). Also on these trials, reactions were faster on Facebook incongruent (*M* = 392.72; *SD* = 40.07) compared to Facebook congruent trials (*M* = 408.26 ms; *SD* = 44.90 ms), *t*(46) = −3.92, *p* < .001, 95% CI [−23.52, −7.57], *d* = 0.57.

### Discussion

We found an attentional bias away from leisure-related items in a sample of students that is overall strongly committed to studying. This was the case even when we analysed attention allocation only on trials with the item participants were strongly attracted to, that is, Facebook. However, when planning to spend more time on leisure-related activities soon after the experiment, more attention was allocated to temptations. In sum, this study provides initial evidence that current motivational states and identification of a self-control conflict shape attention in self-control conflicts. However, our samples might have been particularly strong on goal commitment and self-control (i.e., students with relatively high GPAs at a top tier university). For instance, one could have expected more hours planned for leisure- than studying-related activities during a weekend. Therefore, it is unclear whether the results indeed reflect conflict identification or high habitual self-control. Similarly, we did not find a significant correlation between attention away from temptations and the hours that our participants aimed to spend on studying, maybe because we found overall a very strong attentional bias away from temptations. In the following studies, we therefore manipulated the identification of self-control conflict directly.

## Study 2

Study 2 extended our approach in several ways. First, we directly manipulated the identification of a self-control conflict (or not) to assess whether this evokes different attentional responses to temptations. Further, in both conditions, we reminded participants of their studying goals but manipulated whether using Facebook in conflict or not to this goal. This was possible because the student sample used Facebook both for non-academic goals but also for exchanging study-related information. This way, we could test whether it is the identification of conflict and not the mere activation of a goal that attenuates attention to positive stimuli. We presented only the positive stimulus (i.e., Facebook) as cue in the attention task without the parallel presentation of studying-related items. In the preceding study, it is unclear whether attention is allocated away from positive stimuli or towards studying. We used an even shorter presentation time to tap into earlier attention processes that are hard to control strategically. To the same end, we also changed the location of the cues to be less central and the dependent variable in the attention task to categorization (instead of localization) responses. Finally, we used Facebook as a written word to control for any effect of image saliency. As a neutral comparison cue, we used a letter string that contained the same number of letters.

### Method

#### Participants

One hundred participants (89 women, 8 males, 3 other; *M*_age_ = 20.65, *SD*_age_ = 5.06) from the University of Reading, United Kingdom, participated in exchange for course credit. We calculated that the minimum sample size should be 46 per group at an alpha of .05 and a power of 0.80, and assuming a medium effects size (*d* = 0.50), which was in line with the studies that came closest to the present one (Vogt et al., 2011). Stopping rule was 100 participants because this covered the maximum number of course credits that we were able to award. The study was preregistered before analyses (https://osf.io/rdyqu). Ethical approval was obtained from the School of Psychology and CLS Ethics Committee at the University of Reading for a project called ‘The effects of motivational prioritisation on attention’.

#### Apparatus and Materials

The experiment was programmed and presented using the INQUISIT Millisecond software package (Inquisit 5.0, 2016) on an Intel Core 2 computer with a 75 Hz, 17-inch LDT monitor in a laboratory at the University of Reading.

##### Attention Task

A trial in the attention task started with a fixation screen consisting of a dark blue fixation cross (0.80 × 0.80 cm) on a grey background in the middle of the grey screen. Two white rectangles (13.55 × 9.26 cm) were presented left and right of the fixation cross. The centre of the rectangles was placed 7 cm right and left of fixation. After 500 ms, the word ‘Facebook’ or a letter string in blue composed of the same number of letters appeared for 250 ms in one of the rectangles. A different letter string was used in the practice phase. A probe (: or ..) appeared in one of the rectangles immediately after cue offset. Participants had to categorize the probe by pressing one of two keys (4, 5) on the number pad with their right hand on a QWERTY keyboard. A trial ended after a response was registered or 1,500 ms had elapsed since probe onset. The following trial started after a randomly assigned intertrial interval (ITI) of 300, 600, 1,100 or 1,600 ms. We varied the length of the ITI to keep participants’ concentration high.

We included ‘digit trials’ to reduce strategic monitoring of one area of the screen. On these trials, the fixation cross was followed only by a randomly selected digit for 300 ms in the location of the fixation cross. Participants were instructed to provide the digit presented. The following trials started after 1,100 ms.

#### Procedure

During the entire experiment, participants read the instructions on the screen. Participants were seated approximately 60 cm from the screen. Participants started with a practice phase of the attention task of 36 trials. Hereafter, we induced a no-conflict or conflict mindset in participants. To this end, we told participants that we were interested in learning about their experiences as students. We explained that we study the positive [negative] effects of Facebook and that participants in our studies told us that using [avoiding] Facebook helps them to achieve their study goals. We then asked participants to think about ‘how using [avoiding] Facebook (including Facebook Messenger) helps them to achieve their studying goals’. In order to help participants with answering this question, they were presented with two questions (i.e., no-conflict condition: ‘How does using Facebook help you to gain new energy?’ and ‘How do the people you communicate with via Facebook help you to achieve your studying goals?’; conflict condition: ‘How does using Facebook distract you from studying?’ and ‘How do the people you communicate with via Facebook distract you from studying?’). After answering these questions, participants in both conditions answered a manipulation check question, ‘To what extent does using Facebook help you achieve your studying goals?’ on a 7-point Likert scale (1 = *Not at all* to 7 = *Very much*).

Hereafter, the test phase of the attention task started. The test phase consisted of 87 trials: 40 trials with Facebook as cue, and 40 trials neutral control trials with a letter string as cue, and seven-digit trials. We included control trials for similar reasons as in experiment 1, that is, it allowed us to measure participants’ basic attentional response to cues, which we could use as a baseline. Each cue was presented equally often in the left and right location and predicted the probe location correctly on half of the trials. The two different target stimuli were presented equally often for each cue and in each location. The order of trials was determined randomly and for each participant separately.

After the attention task, we asked participants questions regarding their demographics, usage of Facebook, and studying-related self-regulation patterns. First, we asked them for their gender and age. Then, we asked which year they were studying in and how much they use Facebook (1 = *not at all*; 7 = *5+ times a day*). Afterwards, we asked them to indicate the number of hours they spend on studying during a normal day during the week, how important it is to them to get good marks (1 = *not at all*; 9 = *very much*), and the grade level they are working at. Finally, we asked them for their handedness.

### Results

We excluded the data of one participant because she had made errors on 59.8% trials of the attention task.

#### Manipulation Check

As expected, participants in the conflict identification condition rated Facebook as significantly less useful for achieving their studying goals (*M* = 1.92; *SD* = 1.08) than participants in the no-conflict condition (*M* = 3.38; *SD* = 1.46), *t*(97) = 5.67, *p* < .001, *d* = 0.58.

#### Control Comparisons and Description of the Sample

All participants were highly motivated to achieve good grades (*M* = 7.93; *SD* = 1.05), and this did not differ between conditions, *t*(97) = 0.674, *p* = .50, *d* = 0.07. Four percent of participants reported to work at first-grade level (comparable to As in the US system), 56.6% at 2:1 level (comparable to Bs), 32.3% at 2:2 level (comparable to Cs), and 4% at third grade level (comparable to Ds), making the sample representative of students at the University of Reading. This did not differ between conditions, χ^2^ = 2.54, *p* = .467. Fifty-three percent of participants studied in year 2, 38% in year 1, and 8% in later years, and this was not different between conditions, χ^2^ = 2.265, *p* = .519. Participants indicated to use Facebook relatively often, *M* = 4.93, *SD* = 1.99. Participants in the no-conflict condition report to use Facebook less often, *M* = 4.50, *SD* = 2.02, than participants in the conflict identification condition, *M* = 5.37; *SD* = 1.89, *t*(97) = −2.20, *p* < .05, *d* = 0.22. This is likely an effect of the manipulation (i.e., participants might reflect more critically on their Facebook usage after thinking about how detrimental it is but less after thinking about its usefulness). Nevertheless, the relevant interaction (reported below) involving condition is still significant when controlling for this variable, *F*(1,96) = 5.38, *p* *<* .024, η_*p*_^2^ = 0.053. Participants reported to spend an average of 12.59 hr of a normal weekday on study-related activities, however, we assume that some participants misunderstood the question and reported hours per week as they report more than 24  hours.

#### Main Analyses

Trials with errors were removed (7.36%). We excluded reaction times faster than 150 ms and slower than 1,191 ms, which is the sum of the sample’s mean RT plus three times the sample’s *SD* of the RT (2.29%).

**Figure 3.**
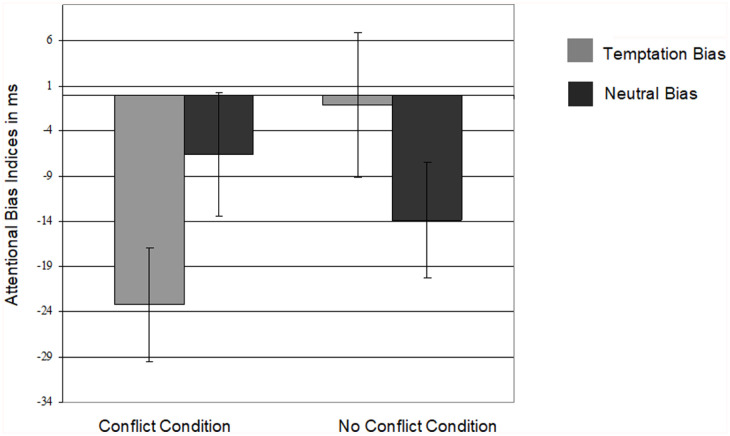
Mean dot probe reaction time difference scores (i.e., temptation/neutral incongruent trials minus temptation/neutral congruent trials) by condition. Error bars represent one standard error of the mean.

To test our hypotheses, we ran an ANOVA with cue validity (valid, invalid), trial type (Facebook, neutral) as within factors and condition (conflict identification, no-conflict identification) as between factors (see also Figure 3). A trial was designated as valid if the probe replaced the cue, and as incongruent if the probe was placed on the other side of the screen. This analysis revealed a main effect of cue validity, *F*(1,97) = 6.49, *p* < .013, η_*p*_^2^ = .063. Importantly, the expected interaction between cue validity, content, and condition was significant, *F*(1,97) = 6.40, *p* < .014, η_*p*_^2^ = 0.062. All other effects were not significant, *F*s <1.

To explore this interaction, we conducted an ANOVA with cue validity (valid, invalid) and trial type (Facebook, neutral) as within factors per condition. In the *conflict identification* condition, we found a main effect of cue validity, *F*(1,48) = 7.83, *p* < .01, η_*p*_^2^ = .140. Importantly, the expected interaction between cue validity and content was significant, *F*(1,48) = 4.67, *p* = .036, η_*p*_^2^ = .089. All other effects, *F*s <1. To further explore this effect, we calculated an attentional bias index by subtracting reaction times on valid trials from reaction times on invalid trials per trial type. Positive indices indicate attention towards cues, negative indices indicate attention away from them. Importantly, the attentional bias index for Facebook was significantly more negative than the index for neutral items, *t*(48) = −2.16, *p* < .04, 95% CI [−32.19, −1.16], *d* = 0.31. This means that participants in the conflict identification condition attended away from Facebook items. There were no significant effects in the no-conflict condition, *F*s <2.17.

### Discussion

When observers perceive a positive stimulus as conflicting with a higher-order goal, attention is shifted away from temptations. In the no-conflict condition, we did not find attention away from Facebook. The results illustrate how different motivational mindsets about the very same positive stimulus cause differential attention allocation patterns. Importantly, these results could explain why attentional bias to positive stimuli appear to be less stable and ‘trait’ like even when observers in general are committed to a goal or are attracted by a temptation. However, we did not find that attention was allocated to Facebook in the no conflict condition. This could be due to participants being less likely to endorse that Facebook is useful to them with an average rating of only 3.38 on a 7-point Likert scale. This will be addressed in the following study.

## Study 3

In the third experiment, we created new positive stimuli by associating neutral stimuli, that is, letters, with symbolic reward in a game-like task. This allowed us to test attention allocation to stimuli that have a clear and unambiguous positive value. For instance, problems to evoke attentional bias to positive stimuli in the preceding study might be due to the various associations that these stimuli might evoke in participants. Furthermore, it might be hard for participants to *not* activate self-control when seeing a temptation such as Facebook in a university setting. Importantly, information related to winning money or symbolic rewards in a game attracts attention (Pearson et al., 2022; Roper & Vecera, 2016; Walsh et al., 2021; Watson et al., 2019) even when reward is no longer administered leading to arguments that attention to such reward-related stimuli is automatic and represents the basis of self-control problems (Watson et al., 2019).

Crucially, we tested whether such presumably overlearned and automatic attentional responses to stimuli that are associated with reward can be attenuated when participants *temporarily* learn that rewarding stimuli are detrimental for the pursuit of a current and prioritized goal (i.e., winning tokens). To this end, participants learned to associate two different letters (‘E’ and ‘F’) with a reward in a game-like task that was performed before and alternating with the attentional measure. We measured attention to both letters and control letters in the attention task. Critically, after having learned the initial reward association, participants learned that in different phases of the experiment only one letter would lead to winning tokens (i.e., ‘active’ reward) in this game whereas reactions to the other (i.e., ‘inactive’, conflicting reward) would lead to the loss of tokens creating interference and conflict with the overall task goal. Thus, the two stimuli represented sub-goals to the higher goal of winning the maximum amount of tokens (cf. Kung & Scholer, 2021), this means, the inactive reward was *in conflict with the overall task goal of winning tokens.* Importantly, they would very frequently switch between these phases. As a consequence, participants had to switch their perception of the letters continuously depending on the phase in the experiment.

The specific rules were not repeated but only indicated by the colour of frames on the screen forcing participants to keep the reward association of both stimuli accessible. This way, we could test whether it was participants’ perception of a letter being conflicting (or not) with a goal that guides attention. Additionally, this aspect of the design would prevent participants from forgetting about the reward association of this stimulus. This experiment thus also allowed us to test whether participants can flexibly switch attention allocation to active and inactive, conflicting rewarding stimuli in the experimental situation using a within-design.

### Method

#### Participants

Forty-nine undergraduate students at the University of Reading, UK, participated in this experiment in exchange for course credits. We calculated that the minimum sample size should be 46 at an alpha of .05 and a power of 0.80, and assumed a medium effect size (*d* = 0.50), which was in line with the studies that came closest to the present one (Vogt et al., 2011). Stopping rule was the end of the term. This study was approved by the Ethics Committee of the School of Psychology and CLS Ethics Committee at the University of Reading for a project called ‘The effects of motivational prioritisation on attention’.

#### Apparatus and Materials

The experiment was programmed using the INQUISIT Millisecond software package (Inquisit 5.0, 2016) on an Intel Core 2 computer with a 75 Hz, 17-inch LDT monitor. All stimuli were presented against a black background.

##### Overview of Tasks

We used a simple task to implement a goal and create rewarding stimuli. In each trial of this task, a single letter appeared briefly on the screen. Participants were instructed to respond by pressing the spacebar when one of two letters was presented. Correct reactions were rewarded with tokens (i.e., symbolic points), and participants were instructed to strive for the maximum score on this task.

More importantly, after learning and practising the basic setup of the task, participants learned that correct reactions to the letters were rewarded depending on specific rules. Specifically, they learned that one of two different colour frames would be presented on the screen. The colour of the frame would indicate that reactions to one letter would lead to winning of tokens (i.e., ‘active’ reward) in the game task performed in parallel to the attention task whereas reactions to the other would cause losing tokens (i.e., conflicting, ‘inactive’ reward). For instance, for some participants, in a phase with a blue frame, ‘E’ would be the active reward that required reactions, whereas ‘F’ would be the inactive reward. Conversely, for these participants, ‘F’ would be the active reward in a phase with a green frame whereas ‘E’ would be the inactive, conflicting reward in this phase.

Letters and control stimuli were also shown in the attention task to examine the allocation of spatial attention. This procedure allowed us to measure attentional processing of reward-related stimuli while participants were simultaneously pursuing the goal. However, because tokens could not be won in the attention task, attending to the letters in the attention task was neither required nor instrumental for the achievement of the goals.

##### Attention and Inducer Task

**Figure 4.**
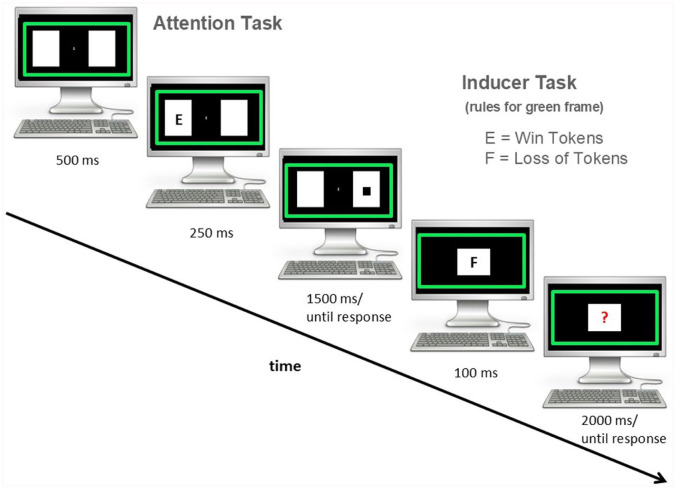
Schematic overview of a combined trial. A trial started with the presentation of a fixation screen for 500 ms, followed by the presentation of a letter for 250 ms. Then a probe (black square) was presented. Participants had to indicate the location of the probe. The attention task trial ended after a response was registered or 1,500 ms had elapsed since the onset of the probe. Every third trial of the attention task was followed by a trial of the inducer task where a single letter or sym was presented for 100 ms. Then a red question mark was presented for 2,000 ms or until a response was registered. Depending on the colour of the frame presented on the screen, participants had to react after E or F to win tokens; the assignment of rules to colours was counterbalanced between participants.

A trial in the attention task started with the presentation of a black fixation cross (5 mm high) in a white square in the middle of the screen (see Figure 4). Along with the fixation cross, two white rectangles (5 cm high × 6 cm wide) were presented at the left and right side of the fixation cross for 500 ms. The middle of each of these peripheral rectangles was 4.6 cm from the fixation cross. Cues and probes were presented within the rectangles. The fixation cross remained on the screen throughout a trial of the dot probe task. A cue (i.e., a letter) appeared for 250 ms. Immediately after offset of the cue, a probe consisting of a black square (0.5 × 0.5 cm) appeared. Responses required locating the probe by pressing one of two keys on the number pad with their right hand. A trial ended after a response was registered or 1,500 ms had elapsed since the onset of the probe. The following trials would start after 400 ms.

After every third trial in the attention task, a trial of the inducer task would start after a 700 ms ITI. A trial would begin with the appearance of a stimulus in the middle of the screen for 100 ms, after which it was replaced by a red question mark (8 mm high). A trial ended with a response (pressing the spacebar, only required for the goal-relevant, reward-related letter) or when 2,000 ms had elapsed since the onset of the question mark (Figure 4). Correct reactions to the active reward were followed by a feedback screen indicating that tokens were won. Incorrect reactions were followed by feedback that either said error (after reactions to neutral or filler stimuli), that tokens were lost (after reactions to the conflicting, currently not relevant letter), or not won (after no reaction to the relevant letter). Feedback was presented for 200 ms. The next trial of the attention task started after 700 ms.

#### Procedure

Participants were seated approximately 60 cm from a computer screen. Instructions were presented on the screen. For the attention task, we asked participants to maintain attention on the fixation cross and to respond as quickly and as accurately as possible to the probe location. We informed them that after responding to the probe, a single letter or symbol (i.e., filler stimuli) would be presented in the middle of the screen. If the letter in the middle of the screen was one of the two goal-relevant, reward-related letters, they should press the spacebar with their left hand when the question mark appeared. Instructions for the inducer task further stated that speed is not important in this task. Participants first practised the attention task in 16 trials, the inducer task in 10 trials, and the combined procedure in a further 21 trials.

Before the test phase, participants were shown the reward-related and control letters and were told that these would be used in the test phase. Specifically, they were told that a blue or green frame would also appear on the screen. Importantly, depending on the colour, reacting after one letter would lead to winning of tokens but reacting after the other would mean to lose tokens. Instructions were not repeated during the phases forcing participants to keep them accessible. Participants were instructed to strive for the maximum score on this task. To make sure that participants picked up this information, they had to repeat it by typing it in. They received feedback whether they were correct.

The attention task consisted of 256 trials, and the inducer task of 80 trials. Each of the possible four types of attention trials (active reward trial; conflicting, inactive reward trials; two types of control trials) was presented 64 times. Letters representing active or conflicting, inactive rewards were ‘E’ and ‘F’. Control letters were the letters ‘O’ and ‘D’. Active reward trials were those in which either ‘E’ or ‘F’ was presented during a phase of the experiment where responses to these letters in the inducer task were rewarded with tokens. In contrast, conflicting, inactive reward trials were trials in phases where either ‘E’ or ‘F’ were shown and reaction to these letters in the inducer task led to the loss of tokens. It was counterbalanced between participants how letters were assigned to colours and rules (e.g., whether ‘E’ [‘F’] was linked to winning tokens with a green [blue] frame). We counterbalanced which coloured frame appeared first in the experiment (i.e., green or blue).

Each cue letter was presented on half of the trials in the left location and on the other half in the right location. Each cue predicted the target location correctly on half of the trials. In the inducer task, each reward-related (e.g., E, F) and control letter (O, D) was presented 16 times. On the remaining 16 trials, a filler stimulus was presented. The order of the trials of both tasks was determined randomly for each participant. The order of the attention task and inducer task trials was determined independently. Hence, the cues that were presented in an attention trial were not predictive of the stimulus that would appear in a consequent trial of the inducer task.

### Results

Participants made errors on 8.2% of trials of the inducer task. We did not further analyse results from this task. For the attention task, trials with errors were removed (5%). We excluded reaction times faster than 150 ms and slower than 885 ms, which is the sum of the sample’s mean RT plus three times the sample’s *SD* of the RT (3.01%).

**Figure 5.**
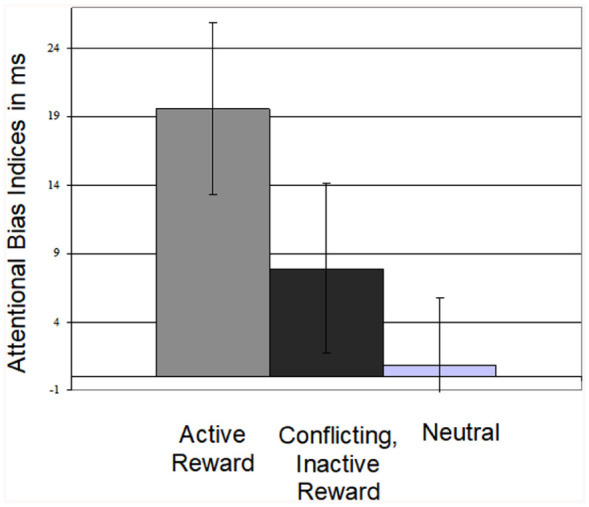
Mean dot probe reaction time difference scores (i.e., reward/neutral incongruent trials minus reward/neutral congruent trials) by condition. Error bars represent one standard error of the mean.

To test our hypotheses, we ran an ANOVA with cue validity (valid, invalid), trial type (active reward, conflicting, inactive reward, neutral) as within factors (Figure 5). A trial was designated as valid if the probe replaced the cue, and as incongruent if the probe was placed on the other side of the screen. The main effect of cue validity approached significance, *F*(1,48) = 3.43, *p* = .070, η_*p*_^2^ = .067. The main effect of trial type was significant, *F*(2,47) = 14.73, *p* < .001, η_*p*_^2^ = .385. Importantly, the expected interaction between cue validity and trial type was significant, *F*(1,47) = 7.52, *p* < .02, η_*p*_^2^ = .241.

To explore this interaction, we calculated an attentional bias index by subtracting reaction times on valid trials from reaction times on invalid trials per trial type. Positive indices indicate attention towards cues, negative indices indicate attention away from them. Importantly, the attentional bias index for active rewards was significantly higher than the index for neutral items, *t*(48) = 3.85, *p* < .001, 95% CI [8.99, 28.63], *d* = 0.55, and importantly, also higher than reward on inactive, conflicting reward trials, *t*(48) = 2.39, *p* < .03 [1.87, 21.54], *d* = 0.34. The difference between the index for inactive, conflicting rewards and neutral items was not significant, *t*(48) = 1.39, *p* < .172 [−3.17, 17.37], *d* = 0.20.

### Discussion

We found different patterns of attention allocation to rewards that are currently rewarded and serve goal pursuit and stimuli associated with reward but not currently rewarded and in conflict with the overall task goal of winning tokens. Specifically, participants paid more attention to stimuli that are related to a current reward than to neutral and, importantly, stimuli related to conflicting, inactive reward.

Importantly, one could argue that responses on this task are influenced by the secondary task that might have functioned as a go-no go task and thus facilitated or inhibited motor responses to stimuli (Dodwell et al., 2024; Hong et al., 2017). We hope that future research will address this concern; however, recent replications of our work suggest that motor responses do not appear to underlie the attentional effects (e.g., Godara et al., 2020; Wentura & Wirth, 2024).

## General Discussion

The present studies investigated whether people’s current motivation to pursue a positive stimulus or activity and specifically whether they perceive it to be in conflict with a higher order goal impacts attentional bias to positive stimuli representing temptations or rewards. Various positive stimuli such as temptations like high-caloric food (Papies et al., 2008; Werthmann et al., 2016) and drug-related cues (Field et al., 2016), or highly liked activities (Roskos-Ewoldsen & Fazio, 1992) attract attention. Therefore, it has been argued that attentional bias to such stimuli is uncontrollable and forms the base of self-control problems (Field et al., 2016; Van Dillen et al., 2013; Werthmann et al., 2016). However, failed attempts to find such biases challenge the assumption that reward attracts attention inevitably (see Hardman et al., 2021; Werthmann et al., 2016). Here, we argued that attention towards positive, tempting items will be attenuated when observers perceive them to conflict with a more important goal. In study 1, we tested attention allocation to images representing study-related items when presented in parallel with leisure-related activities (e.g., YouTube). In this study (and a pilot study), we found that participants attended away from temptations. We also found first evidence that the bias is dependent on whether people perceive a conflict between positive stimuli and more important goals as participants planning to spend more time on leisure activities in the first weekend after the study displayed more attention to temptations. In study 2, we measured attention to a common temptation for students, that is, Facebook, and directly manipulated whether participants perceive Facebook as conflicting or not with their studying goals before the attentional measure. This allowed us to test whether the perception of conflict and not the mere activation or commitment to a higher order goal or simply high self-control aptitude impacts attention allocation. Indeed, only when considering ‘Facebook’ as conflicting, participants attended away from it. In study 3, we tested whether participants can flexibly switch attention allocation to positive stimuli associated with reward depending on whether they are perceived as detrimental to goal pursuit in a within-subjects design. This allowed us to test whether attention is allocated to reward-related stimuli in a flexible way even in the same experimental situation. We found that attention allocation to the active reward, however, attention to conflicting, inactive reward did not differ from a neutral baseline. In sum, this paper reveals how visual attention is allocated when events relevant to conflicting goals are presented in people’s environment. Specifically, in self-control dilemmas, people are inattentive to temptations when conflict is salient.

Our data highlight how self-control regulatory processes shape attention to positive stimuli. Specifically, we show how people’s cognitions about the relation between positive stimuli and a goal cause attention allocation to positive stimuli to vary and, importantly, how considering a conflict between a goal and a positive stimulus can attenuate attention to positive events. The current data therefore support a depiction of attention allocation as a flexible, context-dependent process and contradict models that characterize emotional or motivated attention as stable, inevitable, and thus fully automatic (see Abado et al., 2020; Pool et al., 2016; Vogt et al., 2020; Yiend, 2010, for overviews). As a consequence, our approach helps to explain why attentional bias to positive stimuli vary even within the same person supporting views that attention allocation reflects state rather than trait motivational processes (cf. Hardman et al., 2021; Wiers et al., 2020).

Our findings highlight that recognizing a conflict will not always lead to ‘complete’ avoidance of positive stimuli as in studies 1 and 2. For instance, in study 3, where people cannot forget or completely inhibit the association with reward of the inactive goal, we find attenuated attention to this goal but not attentional avoidance. This might be unsurprising as participants had to remember and to react to this stimulus again. We also forced them to switch very often between the two different task rules without being allowed to forget or inhibit the opposing association. It is therefore likely that when, as in study 2, people can consider a temptation detrimental over the entire experimental session, avoidance will emerge. In contrast, attention will only be attenuated when positive, rewarding stimuli are not only detrimental. This way, study 3 might illustrate the limits of top-down control of attention. Alternatively, it might reflect the very nature of top-down attentional control. For instance, Bundesen (1990) suggested that attention allocation is guided by attentional weights given to stimuli based on, for instance, top-down factors such as goals. It could thus be that this attentional weight differs depending on a stimulus’ varying or stable (ir)relevance in the experimental situation. Related to this issue, it is also important to acknowledge that the conflicts implemented in the different experiment could arguably be considered different (see Kung & Scholer, 2021, for a classification of goal conflicts). Though all conflicts presented inherent goal conflicts, the conflicts, for instance, in studies 1 and 2 might have been more relatable and therefore salient to participants with subsequent consequences for attention in comparison to the conflict that we created in study 3. Further, some have also argued that positive affect (as evoked by the temptations in studies 1 and 2) might impact cognitive processes differently than reward as in study 3 (Chiew, 2021).

Importantly, our data highlight how the identification of a self-control conflict is a relevant process in self-control attempts. Indeed, identification of a conflict is sufficient to find attentional avoidance or attenuation of positive stimuli such as temptations. As a consequence, our findings extend previous research that illustrated how conflict identification impacts the evaluation of and behaviour towards temptations (e.g., Fishbach & Zhang, 2008). Importantly, the current data show that identification of a self-control conflict already influences early, automatic and thus harder to control reactions such as the allocation of attention. Self-control success might thus be less dependent on the ability to inhibit (cf. Katzir et al., 2021; Werner et al., 2022) but rather the ability to see when an action or item is detrimental to one’s goals. This also corresponds with models of self-control that emphasize how metacognitive knowledge on how to recognize and respond to self-control conflicts guides people’s behaviour in self-control processes flexibly and context dependent (Bürgler et al., 2021; Hennecke & Bürgler, 2023).

Understanding of these processes will permit to inform interventions. Our results highlight the importance of understanding motivational mindsets, beliefs and cognitions that cause or prevent conflict identification and their implications for (mal)adaptive attentional bias to positive events, especially in order to influence or, where necessary, to change them for good. This is in contrast to predominant accounts that focus interventions on training observers to acquire an attentional bias away from positive stimuli (e.g., Mellentin et al., 2021) or training people’s ability to exert willpower and ‘resist’ (see Friese et al., 2017, for a review). However, if attentional bias or any self-control process is dependent on beliefs and highly context-dependent cognitions, this does not make sense, and could even be maladaptive. As a consequence, only when training takes beliefs and contextual factors into account can they be efficiently transferrable to relevant real-life situations. For instance, it might be necessary to train people to attend away from positive stimuli in response to the specific situation or mindsets that usually evoke a dysfunctional attentional bias (cf. Salemink et al., 2019; Van Dessel et al., 2023).

Nevertheless, our research is limited in several ways. For instance, we only consider two types of self-control conflict; we therefore hope future research will extend these findings, for instance, to food-related self-control conflicts and attentional bias towards alcoholic beverages (but see Godara et al., 2020; Werthmann et al., 2016). It is also important to replicate our findings, for instance, by implementing a design similar to study 3 but without implementing a stimulus-response conflict at the same time. This research could also address even earlier or unconscious attentional processes. However, the main studies tested relatively early attention allocation; goal-driven attentional processes do also not appear to be limited to later processes of attention (e.g., Forrest et al., 2022; see also Ansorge et al., 2009).

Future research also needs to explore which goals will cause attention *towards* temptations. In our study, Facebook could be realistically described as a tool that allows students to support their studying goals. It is, however, likely that people will sometimes hold false assumptions about the instrumentality of actions or stimuli for their goals. For instance, less health-conscious consumers used eating food that is labelled as healthy as a cue of having made sufficient progress towards health goals and to eat unhealthy food (Finkelstein & Fishbach, 2010). In a similar vein, recent research differentiated between adaptive and maladaptive reasons to indulge in eating high-calorie foods (Prinsen et al., 2019).

We also cannot exclude that there are potential moderators of these effects. For instance, indicators of trait commitment to the goal but also the ability to change the direction of attention (e.g., inhibition) or to switch between different representations and evaluations of stimuli are likely to moderate these effects.

In conclusion, to achieve their goals, individuals need to pay attention to goal-relevant events. Much research has illustrated how goal-relevant events attract attention. In this research, we investigated how attention is allocated when events relevant to conflicting goals are presented in people’s environment. We proposed that early visual processes support the solution of goal conflicts by pointing people to events relevant to prioritized goals and turning them ‘blind’ towards interfering temptations. We tested this assumption in the context of self-control dilemmas where temptations conflict with people’s long-term goals. Using measures of automatic visual attention, three experiments showed attention towards positive, rewarding stimuli can be attenuated. Importantly, self-control conflict needed to be salient and identified to find attentional attenuation to temptations.
